# Environmental activity-based protein profiling for function-driven enzyme discovery from natural communities

**DOI:** 10.1186/s40793-024-00577-2

**Published:** 2024-06-03

**Authors:** Sabrina Ninck, Thomas Klaus, Tatiana V. Kochetkova, Sarah P. Esser, Leonard Sewald, Farnusch Kaschani, Christopher Bräsen, Alexander J. Probst, Ilya V. Kublanov, Bettina Siebers, Markus Kaiser

**Affiliations:** 1 Chemical Biology, Centre of Medical Biotechnology (ZMB), Faculty of Biology, University of Duisburg-Essen, Universitätsstr. 2, 45117 Essen, Germany; 2 Molecular Enzyme Technology and Biochemistry, Environmental Microbiology and Biotechnology (EMB), Centre for Water and Environmental Research (CWE), Faculty of Chemistry, University of Duisburg-Essen, Universitätsstr. 5, 45117 Essen, Germany; 3 Winogradsky Institute of Microbiology, Research Center of Biotechnology, Russian Academy of Sciences, Prospekt 60-Let Oktyabrya 7-2, Moscow, 117312 Russia; 4 Environmental Metagenomics, Research Centre One Health Ruhr of the University Alliance Ruhr, Faculty of Chemistry, University of Duisburg-Essen, Universitätsstr. 5, 45117 Essen, Germany; 5 Centre for Water and Environmental Research (CWE), University of Duisburg-Essen, Universitätsstr. 2, 45117 Essen, Germany; 6 Centre of Medical Biotechnology (ZMB), University of Duisburg-Essen, Universitätsstr. 2, 45117 Essen, Germany

**Keywords:** Activity-based protein profiling, Click chemistry, Chemical proteomics, Environmental microbial communities, Hot springs, Metagenomics, Metaproteomics, Serine hydrolases, Target identification

## Abstract

**Background:**

Microbial communities are important drivers of global biogeochemical cycles, xenobiotic detoxification, as well as organic matter decomposition. Their major metabolic role in ecosystem functioning is ensured by a unique set of enzymes, providing a tremendous yet mostly hidden enzymatic potential. Exploring this enzymatic repertoire is therefore not only relevant for a better understanding of how microorganisms function in their natural environment, and thus for ecological research, but further turns microbial communities, in particular from extreme habitats, into a valuable resource for the discovery of novel enzymes with potential applications in biotechnology. Different strategies for their uncovering such as bioprospecting, which relies mainly on metagenomic approaches in combination with sequence-based bioinformatic analyses, have emerged; yet accurate function prediction of their proteomes and deciphering the in vivo activity of an enzyme remains challenging.

**Results:**

Here, we present environmental activity-based protein profiling (eABPP), a multi-omics approach that extends genome-resolved metagenomics with mass spectrometry-based ABPP. This combination allows direct profiling of environmental community samples in their native habitat and the identification of active enzymes based on their function, even without sequence or structural homologies to annotated enzyme families. eABPP thus bridges the gap between environmental genomics, correct function annotation, and in vivo enzyme activity. As a showcase, we report the successful identification of active thermostable serine hydrolases from eABPP of natural microbial communities from two independent hot springs in Kamchatka, Russia.

**Conclusions:**

By reporting enzyme activities within an ecosystem in their native state, we anticipate that eABPP will not only advance current methodological approaches to sequence homology-guided enzyme discovery from environmental ecosystems for subsequent biocatalyst development but also contributes to the ecological investigation of microbial community interactions by dissecting their underlying molecular mechanisms.

**Supplementary Information:**

The online version contains supplementary material available at 10.1186/s40793-024-00577-2.

## Introduction

Microbial organisms constitute the vast majority of unexplored natural biodiversity and successfully colonize all of the earth’s conceivable ecological niches, thereby forming microbial communities of distinct complexity and fluctuating composition [1–6]. Their ability to thrive under specific conditions, especially in ‘extreme’ ecosystems such as hot springs, is ensured by a unique, often still unexplored enzyme repertoire that turns them into a promising resource for identifying novel (thermostable) enzymes for biotechnological applications [7]. The systematic bioprospecting of microorganisms or microbial communities, sometimes also referred to as environmental biotechnology [3], is frequently based on metagenomic analyses, in particular, if organisms or communities that are non-culturable using standard techniques are screened [8–11]. The identification of promising enzymes for further biocatalyst development from such metagenomic data is then commonly achieved via sequence-driven bioinformatic prediction of protein function [12]. This approach, however, is hampered by a large quantity of proteins of unknown function (i.e., hypotheticals) or misannotated enzymes as well as the presence of large protein superfamilies, for which functional predictions remain difficult [13]. ‘Functional metagenomics’ can help to overcome these limitations by complementing sequence-based approaches with an activity-based screening after the construction of a metagenomic library [14–17]. Although this enables the discovery of novel enzyme classes, it frequently requires elaborate and often challenging cloning and expression efforts with subsequent biochemical characterization [12, 18]. Moreover, this approach delivers no information on the expression and, as valid for all ‘omics’ or phenotype-based next-generation physiology strategies that were invented to unravel the function of a single cell in its native habitat [19], the in vivo activity state of an enzyme-of-interest.

Activity-based protein profiling (ABPP) represents a powerful approach for studying enzyme activities in their native environment and a huge variety of activity-based probes (ABPs) that target different enzymes or even whole enzyme classes are nowadays available [20–22]. A ‘classical’ ABP is composed of a reactive warhead, a tag, and, if present, a chemical or peptidic linker region. While the warhead is often an electrophile-containing inhibitor molecule that targets a nucleophile at the active site of an enzyme for covalent labeling, the tag is used for target detection or enrichment [23]. Thus, ABPP does not only allow the visualization of labeled enzymes via the use of fluorescent reporter groups but also enables target enzyme identification by mass spectrometry (MS) if a moiety for target enrichment, such as biotin, is used as a reporter group [24–26]. Accordingly, although only rarely used in this context, an ABPP experiment with enzyme- or enzyme class-specific ABPs enables a functional annotation of enzymes [27]. With the integration of ‘click chemistry’ into the ABPP workflow, two-step chemical probes have emerged, which facilitate a simple in vivo application of ABPP under physiological conditions [28, 29]. In addition, the invention of affinity-based probes (A*f*BPs) has greatly expanded the enzyme repertoire accessible to ABPP by allowing the use of reversible inhibitors as warheads through the incorporation of a photoreactive group that is activated under UV light to form a covalent bond with a target enzyme [22, 30, 31].

In the last years, ABPP has been mainly used in the context of biomarker or drug discovery as well as in vivo imaging [32–35]; beyond that, diverse applications in microbiology or plant biology, including the study of pathogens or host-pathogen interactions, have been frequently reported [36–38]. Only recently, the use of ABPP in biocatalyst discovery for industrial applications, for example for elucidating lignocellulose-degrading enzymes, has emerged [39–41]. In addition, first reports on the use of ABPP or related approaches for studying microbial communities and isolating functionally active subpopulations, with a focus on host-associated microbial communities, have been made [42–44]. Among these, few studies on the gut microbiome have implemented a combination of ABPP with metagenomics to facilitate the identification of enzymes related to chronic inflammation, drug toxicity, or dietary fiber metabolism in the gastrointestinal tract by employing a metaproteome database that was either constructed from publicly available genomes or self-constructed based on metagenome sequencing [45–47]. ABPP of environmental microbial communities, by contrast, is largely unknown and is usually achieved by profiling isolated strains of environmental microbes rather than by direct profiling of complex communities [48–52]. Recently, activity-based imaging of ammonia- and alkane-oxidizing bacteria in complex microbial communities with the ABP 1,7-octadiyne was reported [53]. This study also used metagenomic sequencing for further confirmation of the functional potential of the targeted microorganisms. However, to the best of our knowledge, no generic workflow for ABPP of microbial communities directly in the environment that also delivers a function-based target identification of the labeled enzymes via downstream MS has been established so far.

We herein describe such an ABPP approach that we refer to as ‘environmental ABPP’ (eABPP). Our approach transfers a combination of ABPP with genome-resolved metagenomics to the field, facilitating the assignment of dedicated activities to enzymes from microorganisms in the environment, even to those that belong to so far uncultured or unknown microorganisms. This has become possible through a tailored sample preparation and data analysis procedure that allows detection of single active enzymes within a complex environmental metaproteome. As a ‘proof-of-concept’, we employed an alkyne-tagged version of the well-established fluorophosphonate-based (FP) ABP [54], which proved to be a versatile probe for the different application types of ABPP. Most importantly, this ABP has been previously shown to allow ABPP of serine hydrolases even under extreme experimental conditions such as elevated temperatures or low pH [52], making it suitable for use in the field. Moreover, employing a broad-spectrum probe increases the number of potentially identifiable target proteins and thus facilitates method validation. Beyond that, members of the serine hydrolase superfamily are widely distributed across all domains of life [55] and are enzymes catalyzing diverse reactions, some of which are of relevance for industrial applications [56–61]. Accordingly, we profiled serine hydrolase activities of microbial communities from two different hot springs located in the Uzon Caldera (Kamchatka Peninsula, Russia) under native conditions directly at the site of sampling. Subsequent biochemical studies then confirmed that this approach can be reliably used for identifying in vivo serine hydrolase activities from environmental community samples.

## Results and discussion

### General workflow design

**Fig. 1:**
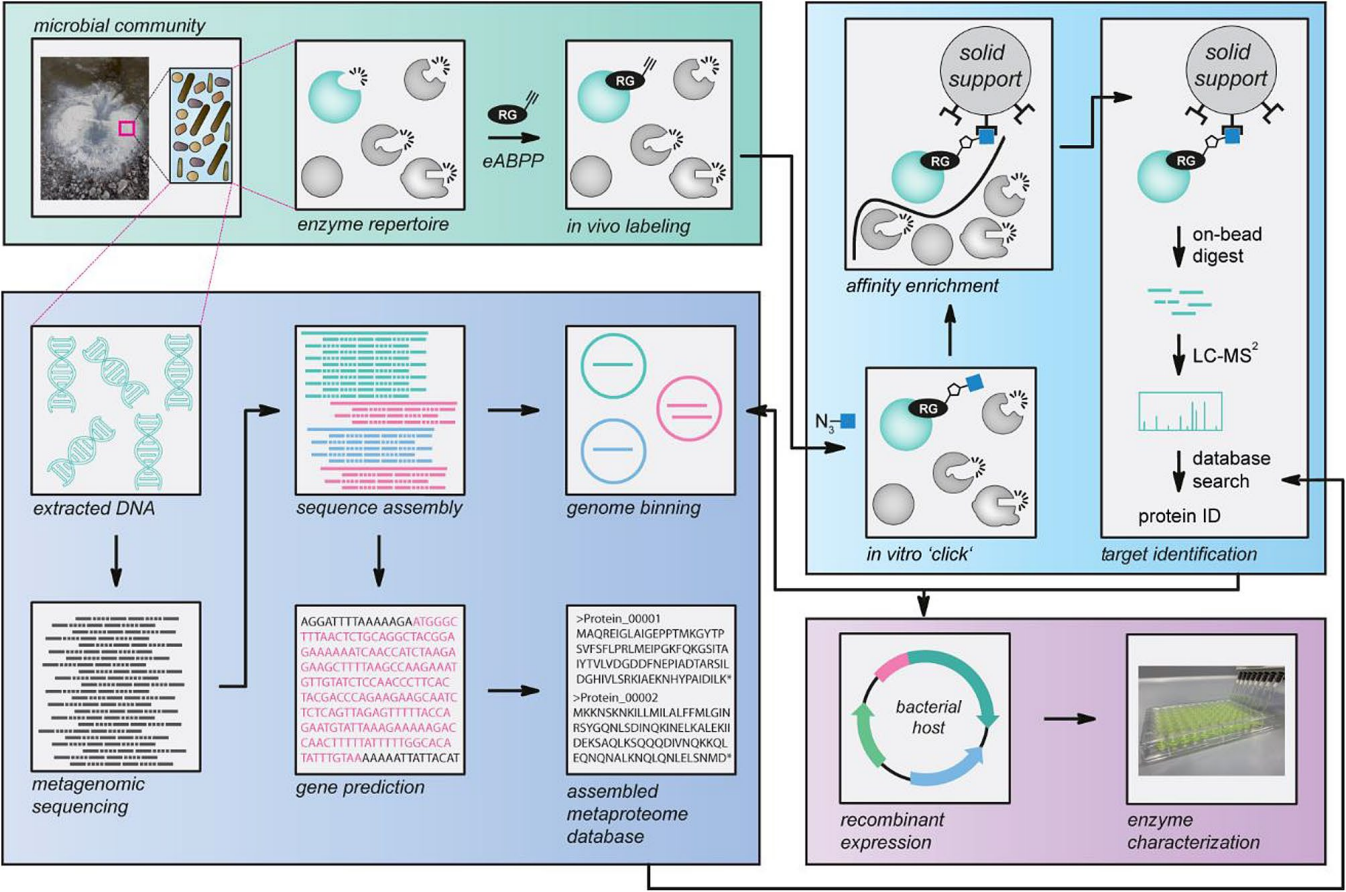
Environmental ABPP workflow. Workflow of the established environmental ABPP (eABPP) approach for the function-based identification of serine hydrolases. This approach can be divided into four different blocks, i.e., sampling and in vivo labeling of an environmental microbial community, metagenomics, target protein identification by LC-MS/MS, and enzyme characterization of a protein of interest

For establishing a function-based eABPP enzyme identification approach of an environmental microbial community, we designed a general workflow based on a combination of ABPP with metagenome sequencing (Fig. 1). This workflow starts with the collection of a microbial community sample, here a hot spring sediment. This sample is then split into seven aliquots and six of them are used for in vivo labeling with an enzyme- or enzyme class-specific two-step ABP; the downstream analysis is thereby focused on active enzymes with the desired function among the microbial enzyme repertoire already at the environmental sampling step. While three aliquots are treated with the respective probe, the other three replicates serve as solvent controls.

For further sample processing including ABP-target enzyme enrichment and MS-based analysis, these in vivo-labeled samples are then transferred to the laboratory along with the remaining sample aliquot. There, the untreated reference sample is used for metagenome analysis. Accordingly, the metagenomic DNA of this sample is extracted and subsequently submitted to metagenome sequencing, enabling the generation of the microbial community-specific metaproteome database via sequence assembly, genome binning, and gene prediction (Supplementary Material 1: Fig. S1). In parallel, the six eABPP samples are processed by protein extraction and two essential clean-up steps including phenol extraction and ammonium acetate precipitation, followed by an in vitro ‘click’ reaction with downstream affinity enrichment of labeled enzymes. The identification of microbial target enzymes is then achieved by on-bead digestion of captured enzymes with downstream LC-MS/MS analysis using the self-assembled in silico metaproteome as a reference database. Finally, the function of eABPP-identified enzymes can be confirmed by recombinant expression of these target proteins and subsequent biochemical enzyme characterization.

Of note, our eABPP approach relies on the availability of ABPs with sufficient target specificity and stability (e.g., for application in hot springs). Moreover, performing ABPP studies in microbial communities has some inherent complexities that require the development of distinct sample preparation procedures for a clean-up of proteins from the complex sample matrix prior to MS analysis, specific data analysis methodologies for detecting the usually only low-abundant single proteins from a metaproteome sample as well as the accurate construction of a metaproteome sequence database [42]. These aspects have already been addressed in prior studies that focused on the gut microbiome and combined ABPP with metagenomics [45–47]. However, profiling environmental communities for the functional identification of active enzymes within an ecosystem implies different prerequisites that are not fully covered by the methods employed in the previous studies. For instance, creating a database from publicly available sequence information, as presented in Mayers et al. [45], would likely suffer from an insufficient representation of the environmental sequences and might increase the chances of false positives due to many irrelevant targets in the database. In contrast to the study of Killinger et al. [46] that also relied on metagenome sequencing, the metaproteome generation in this study only allows the prediction of complete genes that are not gapped by ‘N’s within the assembly (prodigal -m), which results in a highly reliable protein prediction but might reduce the number of identified genes in the protein analysis. Consequently, the herein presented workflow represents the most comprehensive design available to date.

### Microbial diversity of the sampled springs

**Fig. 2:**
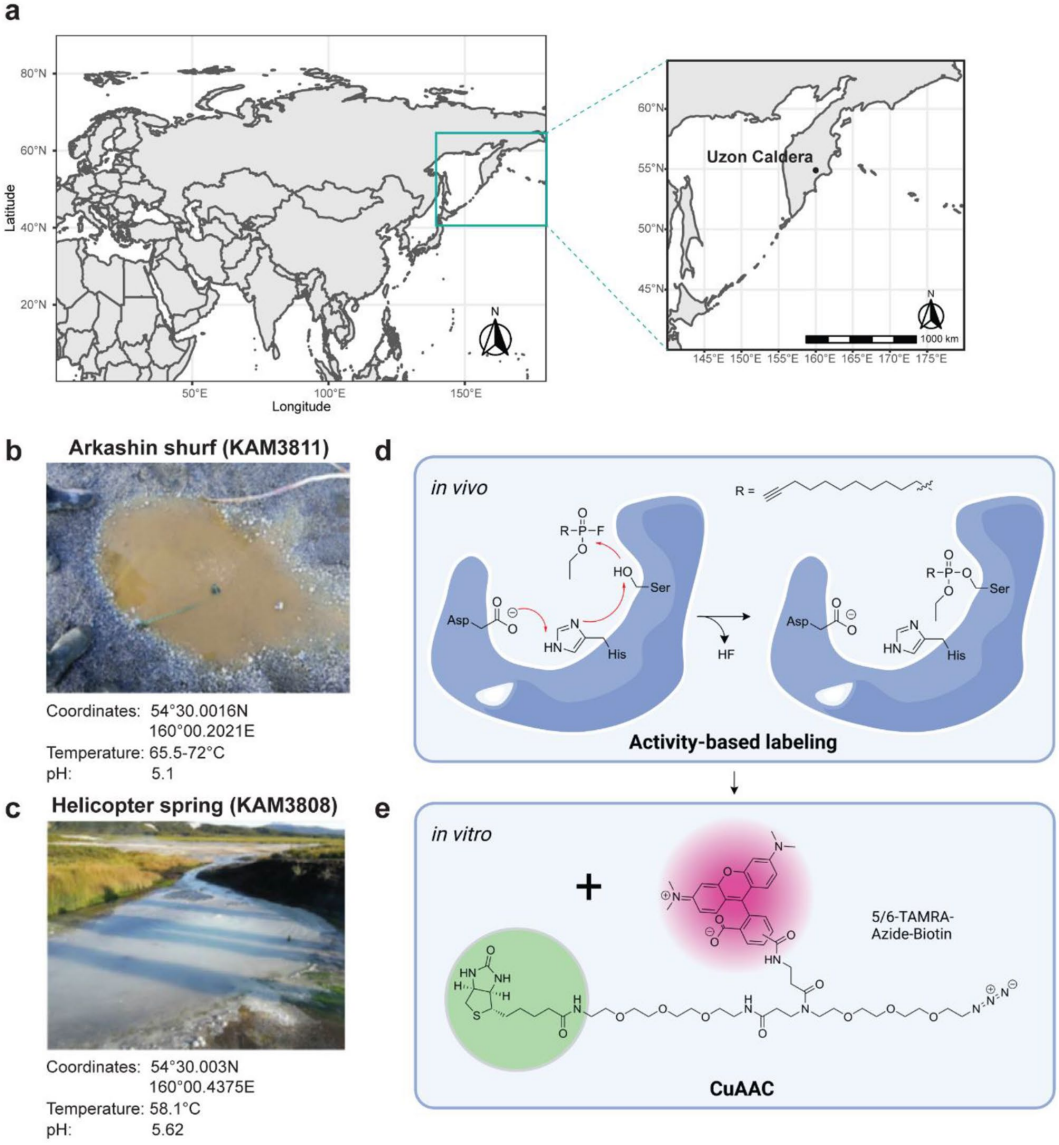
Location of the sampled springs and two-step chemical labeling of serine hydrolases. (**a**) The map shows the location of the two sampled springs ‘Arkashin shurf’ (KAM3811) and ‘Helicopter spring’ (KAM3808) in the Uzon Caldera region, Kamchatka Peninsula, Russia. (**b, c**) A representative picture is shown along with the exact coordinates and physicochemical properties of both springs. (**d**) Chemical structure of the employed FP-alkyne probe and overview of the reaction mechanism taking place at the active site of serine hydrolases during activity-based in vivo labeling of microbial communities from the two springs. (**e**) Attachment of the trifunctional reporter 5/6-TAMRA-azide-biotin via a copper-catalyzed azide-alkyne cycloaddition (CuAAC) for downstream target enzyme enrichment in a second in vitro step after protein extraction from sediments

To showcase the applicability of this eABPP workflow, we sampled environmental microbial communities from two hot springs located in the Uzon Caldera (Kamchatka Peninsula, Russia; Fig. 2a), i.e., the ‘Arkashin shurf’ (KAM3811) and the ‘Helicopter spring’ (KAM3808; Fig. 2b, c) and performed ABPP of serine hydrolases using a FP-based ABP (Fig. 2d, e). These two sites were chosen due to their physicochemical properties (a temperature in the mesothermal range and a slightly acidic pH) that favor the development of an abundant yet unique microbial community. While KAM3811 is an artificial but for more than 30 years stable thermal pool [62] for which 16 metagenome-assembled genomes (MAGs) have been published recently [63], KAM3808 is a larger natural spring with no metagenome data available so far.

**Fig. 3:**
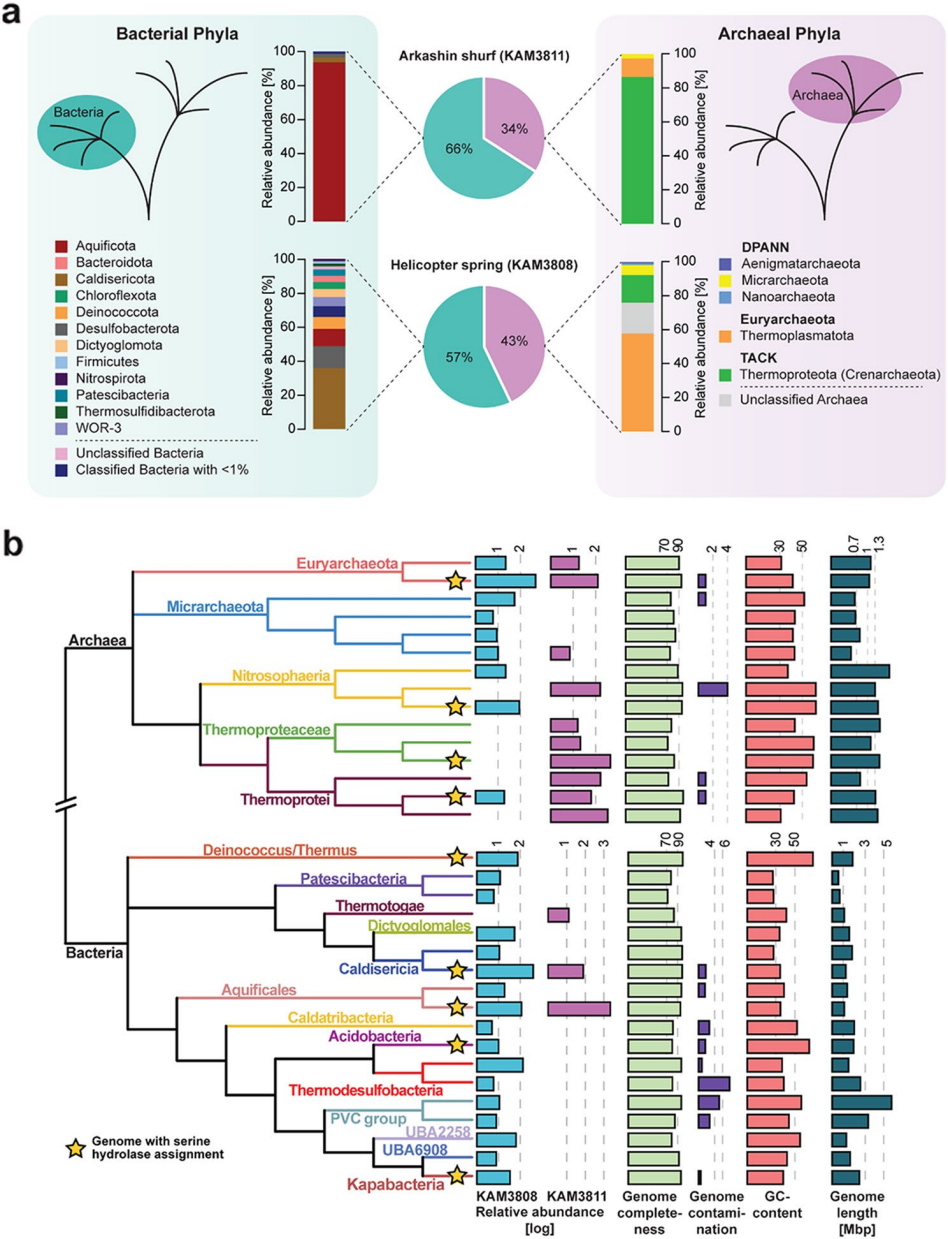
Distribution of microorganisms across KAM3811 and KAM3808. (**a**) The proportion of Bacteria (cyan) and Archaea (magenta) within the sediments sampled for eABPP is depicted as pie diagrams for KAM3811 and KAM3808. An overview of the relative distribution of representative phyla from these domains is given respectively based on the GTDB taxonomy. (**b**) Phylogenetic tree displaying the relationship between the microorganisms found across both springs as calculated with GTDB-Tk based on the dereplicated, binned, and curated metagenomes from KAM3811 and KAM3808. Relative abundances of microorganisms based on the coverage of genomes are given for KAM3811 (magenta) and KAM3808 (light blue), respectively, along with their genome completeness (light green), contamination (purple), GC content (light red), and genome length (dark cyan) as calculated via checkM. The yellow stars indicate the genomes from which predicted serine hydrolases were confidently identified with the applied eABPP approach

All sediment samples (i.e., two times seven aliquots) taken at the two hot springs were treated, processed, and analyzed according to our reported workflow. We then used the metagenome sequence data to gain insights into the community composition of both sediments at the domain and class level by analyzing the relative abundance of ribosomal protein S3 (rpS3) genes within the metagenomic datasets (relative abundance was determined via metagenomic read mapping; Fig. 3a, Supplementary Material 2). The two metagenomes displayed 99.9% and 99.4% of the microbial community of KAM3811 and KAM3808, respectively, with a sequencing effort of 30 Gbp. Both communities showed considerable differences in their distribution between Bacteria and Archaea as well as in the number of different classes present within each domain. For KAM3811, 66% of the assigned species belonged to the bacterial domain, with the Aquificota being the dominating phylum, while the archaeal domain was mainly represented by the Thermoproteota (Supplementary Material 1: Table S1). In the phenotypically and phylogenetically more diverse spring KAM3808, bacterial species account for 57% of all organisms, with the Caldisericota making up the largest fraction, followed by the Desulfobacterota and the Aquificota. In contrast to KAM3811, archaeal species comprised mainly Euryarchaeota, while Archaea from the DPANN group made up the smallest fraction (Supplementary Material 1: Table S2). In a more detailed analysis of these metagenomic datasets, again by relying on genome coverages, all phyla identified within the respective spring were displayed in a domain-specific phylogenetic tree with their corresponding abundancies (Fig. 3b; Supplementary Material 3). For KAM3808, which displayed more heteromorphic sediments, rpS3 genes of 49 microorganisms could be identified, with *Aciduliprofundum* sp. (relative abundance of 488.1), *Caldisericum exile* (408.2) and *Caldimicrobium thiodismutans* (139.9) as the most abundant species (Supplementary Material 1: Table S2). KAM3811, a distinctly smaller thermal pool with homogenous brown to red sediments, comprised 19 different organisms as determined from rpS3 gene analysis. Among them, *Sulfurihydrogenibium* sp. (2329.1) was identified as the dominating genus, followed by *Pyrobaculum ferrireducens* (411.3) and *Caldisphaera* sp. (389.6; Supplementary Material 1: Table S1).

Altogether, these experiments unambiguously show that the sampled communities are highly diverse in their microbial composition and should thus harbor diverse enzymes with serine hydrolase activity. The detected phyla largely comprise unexplored microorganisms with a functional potential yet to be uncovered.

### Multifaceted annotation of serine hydrolases in the metaproteome database

The assembled metagenomes were then used to construct two metaproteome databases consisting of 45,649 (KAM3811) and 99,930 (KAM3808) protein-coding sequences, respectively. We relied on high-quality reads for assembly and only predicted full-length gene sequences (fragments were ignored). Combined with a fairly great sequencing depth, we provide enough coverage for a robust database for protein identification, yet avoiding the usage of public datasets, thereby decreasing the rate of false positives during detection. Annotation of the here-derived metaproteome databases against the UniRef100 database resulted in 35,323 genes encoding proteins with a predicted function for the KAM3811 dataset, while 8,575 proteins remained uncharacterized. Analogously, 85,393 genes encoded proteins with a predicted function for the KAM3808 dataset, whereas 17,923 genes encoded uncharacterized proteins. The discrepancy between the number of proteins with predicted function and hypothetical ones stemmed from the lack of represented similar genes within public databases or from the fragmentation of genes. Within the metaproteome dataset of KAM3811 and KAM3808, this annotation procedure surprisingly led to only two and sixteen proteins, respectively, which were assigned the term ‘serine hydrolase’. This is a consequence of the variety of terms used for describing the different members of the serine hydrolase superfamily. Predicting the actual number of serine hydrolase sequences present in the two datasets thus remains intricate, since it requires elaborate manual curation of relevant search strings. Although this difficulty is also addressed by our function-based eABPP approach, a bioinformatic solution to this problem would be beneficial for future studies.

The advantage of using a metagenome-derived metaproteome database relies on the exact blueprint of the genes and in silico predicted proteins for improved matching of newly generated peptide fragments. Public databases might not have the respective gene from the same organism, i.e., it might have differences in its amino acid sequence though conveying the same function. This might substantially decrease the number of assigned peptide fragments. Secondly, un(der)explored ecosystems often carry novel genes and thus proteins, which are not yet available in public databases and would thus not generate a proper match. In fact, recent analyses of public metagenomes demonstrated a continuous increase of novel protein families across new metagenomes [64, 65]. Thirdly, we resolved the metagenome at the genome level, which enables the direct assignment of enzymes to respective organisms, which is usually not possible with public databases (as general taxonomic assignments of functional genes are not stable in nature due to, e.g., horizontal gene transfer).

### Identification of active serine hydrolases by eABPP

**Fig. 4:**
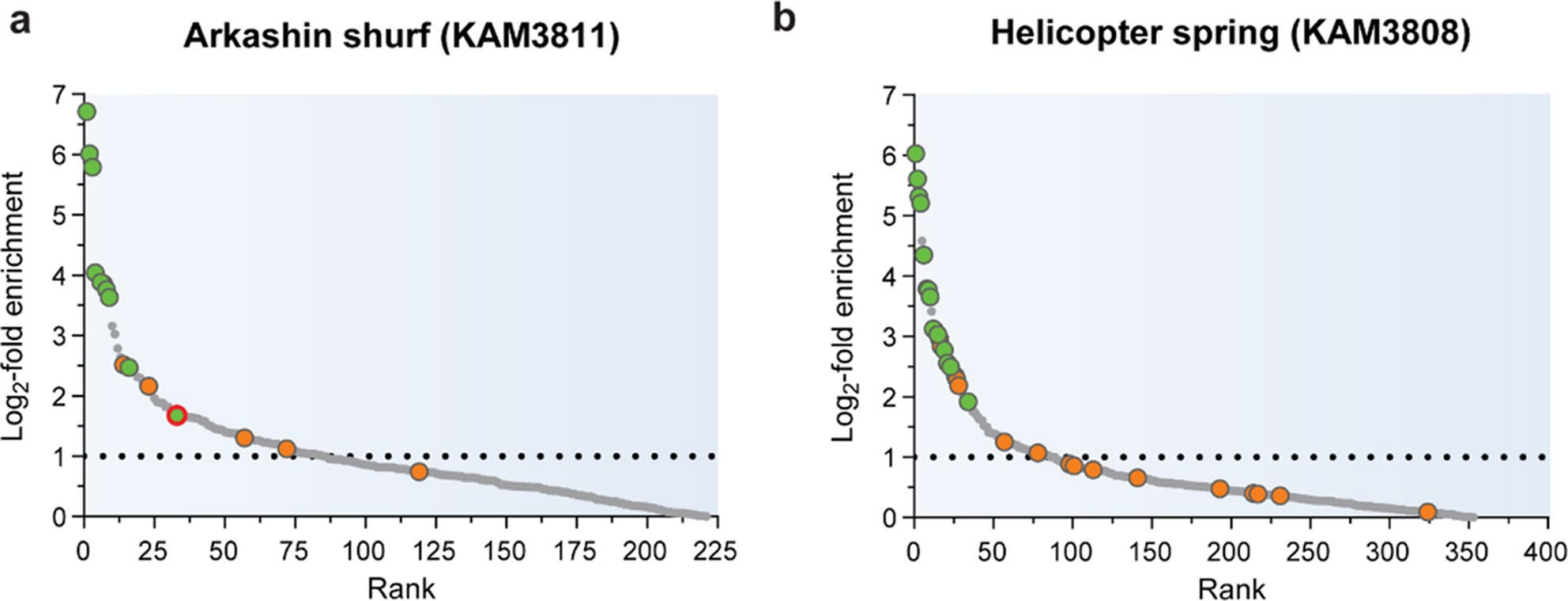
Predicted serine hydrolases identified from the sampled hot springs. Log2-fold enrichment of identified proteins labeled with FP-alkyne compared to the DMSO control for the sediments sampled from (**a**) KAM3811 and (**b**) KAM3808. Proteins predicted as serine hydrolases are displayed as colored dots (green: *p*-value ≤ 0.05, orange: *p*-value ≥ 0.05). The protein chosen for function validation is circled in red. Hits lying above the dotted line were more than two-fold enriched with the probe and were therefore considered primary hits. Each treatment group comprised three biological replicates

To identify active serine hydrolases from the sampled environmental communities, the self-constructed metaproteome databases were then employed as reference databases for the analysis of eABPP MS data resulting from an affinity enrichment of FP-alkyne-labeled target proteins via a biotin handle, on-bead digest, and subsequent LC-MS/MS analysis. Overall, 811 and 1,489 protein groups (comprising proteins that were not distinguishable based on the identified peptides) were identified for the KAM3811 and KAM3808 datasets, respectively, excluding hits from the implemented contaminants database. After filtering the initial data (see the methods section), a total of 385 protein groups for KAM3811 and 672 protein groups for KAM3808 remained for further analysis (Supplementary Material 4). Of these, 1.6% and 2.1% of protein groups for KAM3811 and KAM3808, respectively, comprised more than one protein. For KAM3811, 221 protein groups displayed a positive log2-fold change when comparing the group of FP-labeled samples against the DMSO-treated control group, while KAM3808 harbored 353 log2-fold enriched protein groups (Fig. 4). In contrast to the two large metaproteomes, these strongly reduced protein numbers now allowed a manual curation of serine hydrolase prediction by complementation of the UniRef100-based protein annotation with sequence searches against the Pfam, NCBI CCD and InterPro databases. Where necessary, additional searches against the SWISS-MODEL Template Library or with HHpred were also performed to identify structural homologs (Supplementary Material 5).

For KAM3811, this extended annotation procedure predicted 15 protein groups as serine hydrolases (Table 1). Most notably, these protein groups were predominantly found among the top enriched hits, with 11 protein groups found among the top 30 enriched hits. For KAM3808, which showed a larger microbial diversity, 31 predicted serine hydrolases were obtained (Table 2), with 19 protein groups found among the top 30 enriched hits. Further protein groups found among these top 30 enriched hits either lacked evidence for being potential serine hydrolases or remained uncharacterized as no conclusive information on their function was obtained based on sequence- or structure-based homology. However, their annotation from the UniRef100 database indicates that for both datasets, many of these are potential components of membrane transporters or other membrane-associated proteins (Supplementary Material 4). The top-ranked serine hydrolases likely represent the most active enzymes under the in vivo conditions of the hot springs due to either their higher abundances or their elevated activities compared to other serine hydrolases in the sampled environment. Although lower-ranked serine hydrolases not only feature a smaller fold change but often also a smaller *p*-value, this does not necessarily mean that they are less valid hits. The inhomogeneity of sediment and thus between samples as well as the necessity for imputation of missing data can add a bias to these computed parameters, especially for low abundant or inconsistently detected hits. Another thing to be aware of is that the use of a large metaproteome database increases the likelihood of false positives during peptide spectral matching. eABPP, however, provides a degree of inherent confirmation of correct identification through matching with the profiled function. Please note that we here report only those serine hydrolases as hits for both springs which were confidently identified by such a sequence- or structure-based homology analysis to demonstrate the robustness of the eABPP approach. It is however likely that there are several more enzymes displaying serine hydrolase features and activities across the two sets of enriched proteins that were not uncovered by this analysis.

**Table 1:** Predicted serine hydrolases identified for KAM3811

No.	Log2-fold change	-Log *P*	Identifier	Protein annotation^#^
1	6.711	2.287	ExploCarb_3811S_S4_2994_length_2665_cov_56_1	Peptidase S8, subtilisin-related
2	6.011	4.069	ExploCarb_3811S_S4_103_length_35923_cov_48_7	Penicillin/GL-7-ACA/AHL acylase
3	5.787	3.448	ExploCarb_3811S_S4_37_length_59942_cov_23_2	Peptidase S8, subtilisin-related
4	4.038	2.999	ExploCarb_3811S_S4_179_length_24712_cov_181_27	Penicillin/GL-7-ACA/AHL acylase
6	3.881	3.141	ExploCarb_3811S_S4_477_length_13181_cov_9_10	Peptidase S8, subtilisin-related
7	3.852	0.946	ExploCarb_3811S_S4_782_length_9153_cov_146_4	Penicillin/GL-7-ACA/AHL acylase
8	3.760	1.690	ExploCarb_3811S_S4_412_length_14764_cov_159_5	Peptidase S8, subtilisin-related
9	3.630	2.838	ExploCarb_3811S_S4_5916_length_1495_cov_4_1	Peptidase_S8/S53_dom
14	2.520	1.043	ExploCarb_3811S_S4_1380_length_5439_cov_180_2	Peptidase_S9
16	2.470	3.204	ExploCarb_3811S_S4_7165_length_1270_cov_6_1	Peptidase S8, subtilisin-related
23	2.162	0.734	ExploCarb_3811S_S4_1591_length_4725_cov_42_1	Protein of unknown function DUF915, hydrolase-like
33	1.675	1.544	ExploCarb_3811S_S4_483_length_13114_cov_941_5	Uncharacterised protein family UPF0227/Esterase YqiA
57	1.303	0.869	ExploCarb_3811S_S4_1740_length_4311_cov_157_3	Xaa-Pro-like_dom
72	1.122	0.593	ExploCarb_3811S_S4_259_length_20079_cov_121_4	Peptidase_S49
119	0.745	0.826	ExploCarb_3811S_S4_1428_length_5265_cov_777_6	Peptidase_S49

# InterProScan annotation on either protein family or domain level

**Table 2:** Predicted serine hydrolases identified for KAM3808

No.	Log2-fold change	-Log *P*	Identifier	Protein annotation^#^
1	6.028	2.903	ExploCarb_3808S_S2_15464_length_1283_cov_2_1	GDSL lipase/esterase
2	5.607	1.416	ExploCarb_3808S_S2_19633_length_1026_cov_55_1	Putative S8A family peptidase^†^
3	5.316	3.107	ExploCarb_3808S_S2_5917_length_3034_cov_1_3	Peptidase S8, subtilisin-related
4	5.204	1.675	ExploCarb_3808S_S2_470_length_27453_cov_4_11	SGNH_hydro
6	4.351	3.267	ExploCarb_3808S_S2_593_length_22580_cov_176_21	Esterase/lipase
8	3.791	1.563	ExploCarb_3808S_S2_12785_length_1520_cov_45_2	Peptidase S8, subtilisin-related
9	3.771	2.222	ExploCarb_3808S_S2_10394_length_1832_cov_227_1	Peptidase_S8/S53_dom_sf
10	3.657	2.291	ExploCarb_3808S_S2_1348_length_11661_cov_169_4	PNPLA_dom
12	3.124	2.085	ExploCarb_3808S_S2_277_length_40468_cov_33_33	Peptidase S8A, fervidolysin-like
13	3.101	1.017	ExploCarb_3808S_S2_2184_length_7533_cov_3_4	Penicillin/GL-7-ACA/AHL/aculeacin-A acylase
15	3.030	1.860	ExploCarb_3808S_S2_12072_length_1604_cov_127_1	Peptidase_S8/S53_dom_sf
16	2.954	0.995	ExploCarb_3808S_S2_1901_length_8595_cov_3_5	Penicillin/GL-7-ACA/AHL acylase
17	2.839	1.008	ExploCarb_3808S_S2_5211_length_3405_cov_171_3	Peptidase family S66
19	2.776	2.407	ExploCarb_3808S_S2_84_length_75002_cov_30_5	PNPLA_dom
21	2.560	1.689	ExploCarb_3808S_S2_12016_length_1611_cov_2_1	AB_hydrolase_1
23	2.492	2.063	ExploCarb_3808S_S2_1180_length_13195_cov_3_10	SGNH_hydro_sf
26	2.344	0.874	ExploCarb_3808S_S2_5180_length_3427_cov_2_3	Peptidase S8, subtilisin-related
27	2.282	0.487	ExploCarb_3808S_S2_14352_length_1370_cov_1_2	AB_hydrolase_1
28	2.185	1.070	ExploCarb_3808S_S2_1_length_789533_cov_14_559	Peptidase S8, subtilisin-related
34	1.914	1.385	ExploCarb_3808S_S2_7708_length_2386_cov_3_1	Lipase_bact_N
57	1.253	0.146	ExploCarb_3808S_S2_17847_length_1126_cov_1_2	AB_hydrolase_1
78	1.068	0.956	ExploCarb_3808S_S2_4160_length_4192_cov_19_2	Penicillin/GL-7-ACA/AHL acylase
98	0.884	0.766	ExploCarb_3808S_S2_2577_length_6506_cov_183_2	Peptidase S8, subtilisin-related
101	0.856	0.606	ExploCarb_3808S_S2_5424_length_3285_cov_7_1	Peptidase S8, subtilisin-related
113	0.792	0.541	ExploCarb_3808S_S2_45_length_96604_cov_169_9	PNPLA_dom
141	0.657	0.331	ExploCarb_3808S_S2_593_length_22580_cov_176_11	Peptidase S8, subtilisin-related
193	0.477	0.170	ExploCarb_3808S_S2_1272_length_12299_cov_60_1	AB_hydrolase_1
214	0.397	0.143	ExploCarb_3808S_S2_12631_length_1536_cov_2_2	Peptidase S1C, Do
217	0.387	0.590	ExploCarb_3808S_S2_858_length_17095_cov_20_14	Hydrolase_4 (Serine aminopeptidase, S33)
231	0.358	0.176	ExploCarb_3808S_S2_21_length_132706_cov_167_47	Peptidase S1C
324	0.089	0.091	ExploCarb_3808S_S2_2714_length_6206_cov_211_1	Penicillin/GL-7-ACA/AHL acylase

# InterProScan annotation on either protein family, homologous superfamily or domain level unless stated otherwise

† UniRef100 annotation

The eABPP-enriched active microbial community serine hydrolases comprised enzymes from the entire serine hydrolase superfamily, including proteases (or peptidases as synonymous term), lipases, amidases, and esterases [55]. Across both springs, we found proteins predicted as serine-type peptidases from the (super)families S1 (chymotrypsin family, subfamily S1C), S8/S53 (clan SB: S8 (subtilisin family, including subfamily S8A), S53 (type peptidase: sedolisin)), S9 (prolyl oligopeptidase family), S15 (type peptidase: Xaa-Pro dipeptidyl peptidase), S33 (type peptidase: prolyl aminopeptidase), S45 (type peptidase: penicillin G acylase precursor), S49 (protease IV family), and S66 (type peptidase: murein tetrapeptidase LD-carboxypeptidase). Moreover, we detected proteins predicted as esterases/lipases, including enzymes from the SGNH hydrolase superfamily or enzymes containing a GDSL, a PNPLA (patatin-like phospholipases), or a Lipase_bact_N domain, and a DUF915 family enzyme with structural similarity to esterases/lipases as well as a putative esterase from the UPF0227 family. Furthermore, enzymes predicted as alpha/beta fold-1 hydrolases without further classification but with esterases/lipases as structural homologs were identified from these datasets.

Altogether, these results show that our eABPP approach enabled the identification of functionally diverse serine hydrolases, ranging from various proteases to esterases/lipases up to serine hydrolases with uncharacterized functions (e.g., DUFs or UPFs), thus being superior over a simple bioinformatic metagenome annotation procedure. Of note, the majority of the identified active serine hydrolases are annotated as subtilases or penicillin acylases, which demonstrates that the applied eABPP screen indeed allows the detection of thermostable representatives of the functionally highly diverse family of serine hydrolases that already find application for industrial purposes [66–69].

### Function validation of a selected serine hydrolase

**Fig. 5:**
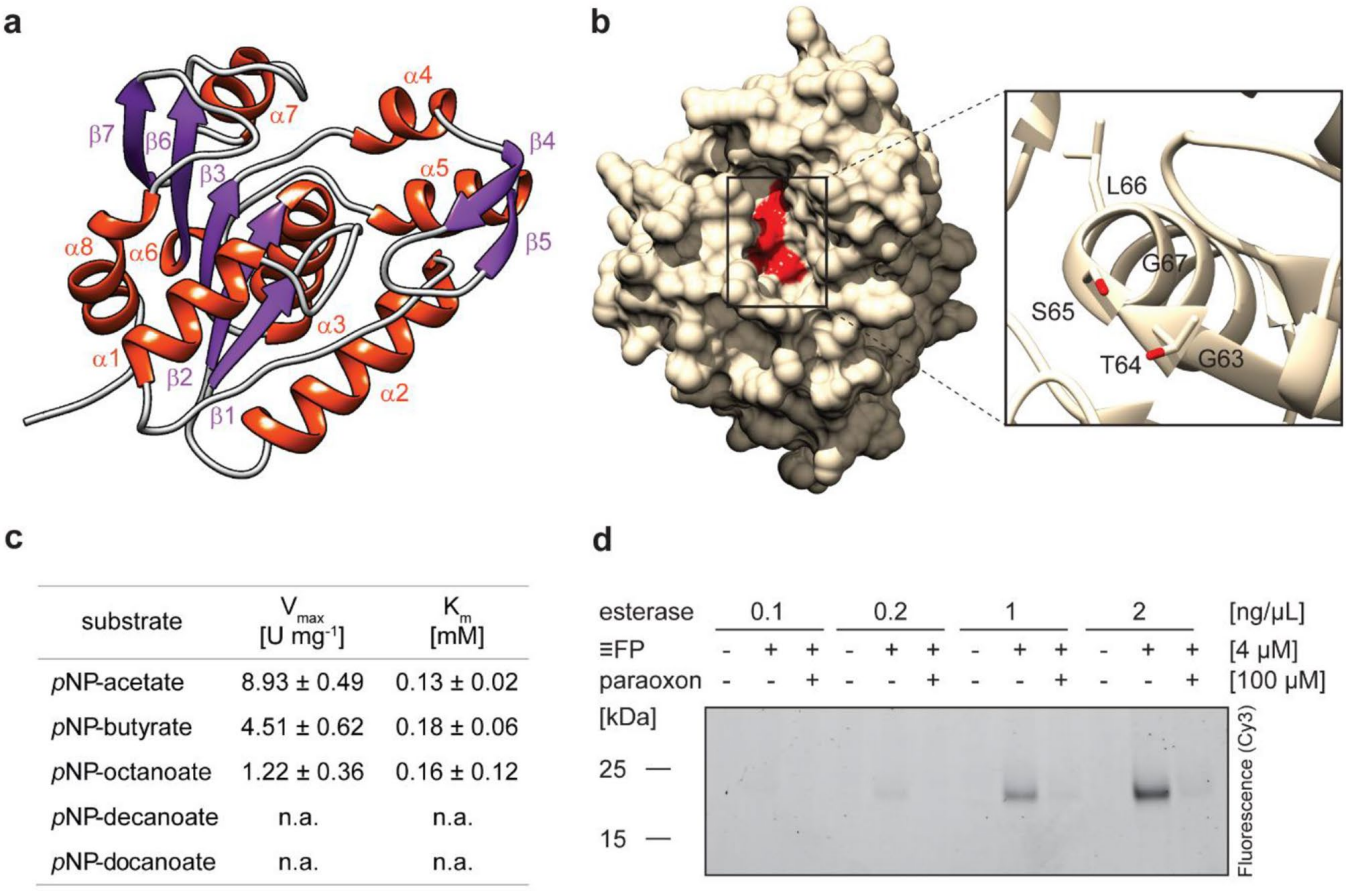
Characterization of the putative esterase. (**a**) Predicted structure of the UPF0227 protein with the secondary structures visualized in red (alpha helices) and purple (beta strands). The five-stranded parallel beta-sheet consists of the strands β1, β2, β3, β6 and β7. (**b**) Surface-displaying structure of the putative esterase. The close-up displays the conserved serine hydrolase motif GxSxG, comprising the residues G63, T64, S65, L66, and G67, which is located in a substrate pocket (depicted in red). Structure prediction was performed with AlphaFold and the output was processed in Chimera. (**c**) Kinetic characterization of the esterase using the *p*NP-substrates *p*NP-acetate, -butyrate, -octanoate, -decanoate, and -dodecanoate at concentrations up to 0.7 mM at pH 8.0 and 70 °C and calculation of V_max_ and K_m_. Values represent the mean of three technical replicates ± SD. (**d**) In vitro labeling of varying amounts of the esterase with FP-alkyne in the presence or absence of paraoxon

Finally, to confirm the suitability of eABPP for a function-based enzyme identification from an environmental microbial community, we chose the serine hydrolase ExploCarb_3811S_S4_483_length_13114_cov_941_5 (Table 1) from our list of eABPP-identified serine hydrolases for further bioinformatical and biochemical characterization. This 191 amino acid-containing enzyme was selected due to its relatively short and complete gene sequence as well as the availability of a straightforward enzyme kinetic assay. In addition, the differential annotation of this enzyme (see Supplementary Material 5) nicely demonstrates the caveats of bioinformatic-based protein function prediction when relying only on a single database, as it was predicted as an esterase based on the UniRef100 database, while the domain-predicting databases Pfam and InterPro revealed it as a UPF0227 (uncharacterized protein family 0227) protein. We therefore generated an AlphaFold-derived structural model for this protein that revealed a fold in which a GTSLG sequence previously associated with thioesterase activity [70] is accommodated at the functionally conserved GxSxG active site motif of serine hydrolases (Fig. 5a, b). The esterase sequence shows 100% identity to a predicted esterase (NCBI accession number: PMP76296.1) from a *Sulfurihydrogenibium* sp. MAG from a published metagenome, originating from the same spring [63]. BLAST-based sequence as well as HHpred-based structural homology searches further confirmed its similarity to other esterases (Supplementary Material 1: Table S3). These analyses together suggest that the UPF0227 domain-containing protein is most likely a serine esterase with however unknown substrate specificity.

In order to biochemically validate the function of the putative esterase, *E. coli* Rosetta(DE3) cells were transformed with the codon-optimized gene sequence cloned into a pET-28b(+) vector for heterologous protein expression. The purified enzyme was biochemically characterized using *para*-nitrophenyl (*p*NP) substrates. The putative esterase showed highest activity at a pH of 8.0 (Supplementary Material 1: Fig. S2a) and a temperature of 70 °C (Supplementary Material 1: Fig. S2b) using *p*NP-butyrate as a substrate. Moreover, enzyme kinetics were determined using different *p*NP-esters (Fig. 5c). Effective hydrolysis was observed for *p*NP-acetate (V_max_ = 8.93 U mg^− 1^, K_m_ = 0.12 mM), *p*NP-butyrate (V_max_ = 4.51 U mg^− 1^, K_m_ = 0.18 mM) and *p*NP-octanoate (V_max_ = 1.22 U mg^− 1^, K_m_ = 0.16 mM), confirming its function as an esterase, whereas no activity was measured for *p*NP-esters with longer chain lengths, such as *p*NP-decanoate or *p*NP-dodecanoate. Furthermore, in vitro ABPP of the purified esterase with FP-alkyne, analogously to the in vivo ABPP experiment, revealed robust labeling over a range of esterase concentrations, which could be strongly diminished by pre-incubation with the generic serine esterase inhibitor paraoxon (Fig. 5d). Consequently, the esterase was proven a bona fide target of the covalent-acting FP probe. In line with this result, FP-alkyne was furthermore shown to reduce the esterase activity of the enzyme in correlation with the applied probe concentration (Supplementary Material 1: Figure S2c).

## Conclusions

In this study, we established eABPP for the efficient function-based identification of active enzymes directly from the environment. The eABPP workflow relies on activity-based labeling of a microbial community sample in combination with metagenome sequencing. In this way, the bioinformatic annotation of the metagenome can be directly confirmed through the activity-dependent reaction of the ABP-targeted enzyme. This provides direct experimental evidence for the bioinformatically predicted enzyme function along with its protein identification. As a showcase, we profiled epi-sedimentary communities from two hot springs located in the Uzon Caldera region of Kamchatka (Russia), exploiting the broad range serine hydrolase target specificity of FP-alkyne for functional enzyme annotation. Thorough bioinformatic analysis of the eABPP-enriched and -identified proteins from the hot spring sediments revealed that the top-ranked hits mainly comprised different serine peptidases, mostly from the families S8 and S15, as well as esterases/lipases, thus demonstrating the applicability of the method. To further corroborate the versatility of our eABPP approach, we heterologously expressed an ‘uncharacterized protein family UPF0227’ enzyme found among the top eABPP-enriched hits and confirmed its esterase activity.

We therefore believe that our approach can be further extended to profile additional enzyme activities of microbial communities from diverse ecosystems, in particular as a large and constantly growing repertoire of probes with a wide variety of warheads for targeting different enzymes or enzyme classes is already available. These probes provide a broad coverage of enzymes from different classes of the Enzyme Commission (EC) number classification system. Although most of the probes have been designed for hydrolases (EC 3), several probes targeting different enzymes from subclasses of the oxidoreductases (EC 1), transferases (EC 2), mainly kinases, and ligases (EC 6) as well as ‘reverse-polarity’ probes engaging protein electrophiles instead of nucleophilic residues, which target enzymes across different classes, have been developed [22, 71, 72]. Despite the broad coverage of enzymes with different modes of action, ABPP is still limited to a few enzyme families in light of the great diversity of existing enzyme activities. As the probes available to date mainly address metabolic enzymes, eABPP may be adapted for ecological research, for instance to study different aspects of ecosystem functioning or to decipher enzyme activity patterns, e.g., from synergistic or mutual community interactions. Besides, we anticipate that our approach may open new avenues in enzyme discovery, especially for finding enzyme activities that harbor potential for application in biotechnological processes. These include, for example, cysteine proteases or glycoside hydrolases, for which a broad set of probes such as the well-established E64- or cyclophellitol-based ABPs, which we believe would also allow reliable enzyme identification in complex environmental samples with a rather low abundance of single proteins, is currently at hand [73, 74]. Glycoside hydrolases, for instance, are of particular interest with regard to biocatalyst discovery since they largely function in (lignocellulosic) biomass degradation [39–41, 75]. In addition, it might be reasonable to design more specific probes with activity towards serine hydrolases that function in the degradation of environmental pollutants (e.g., PETases and different biphenyl and meta-cleavage product hydrolases capable of cleaving mono- and bicyclic aromatic compounds), which would allow a more precise confirmation of function compared to broad-spectrum probes with a large and diverse target repertoire, as these enzymes are of interest for environmental and industrial applications.

### Electronic supplementary material

Below is the link to the electronic supplementary material.

Supplementary Material 1

Supplementary Material 2

Supplementary Material 3

Supplementary Material 4

Supplementary Material 5

## Data Availability

The mass spectrometry proteomics datasets generated during this study have been deposited to the ProteomeXchange Consortium via the PRIDE [76] partner repository (https://www.ebi.ac.uk/pride/archive/) with the project accession number PXD025833. Processed mass spectrometry data is contained in Supplementary Material 4. All raw sequencing data was submitted to the NCBI (https://www.ncbi.nlm.nih.gov/) Sequence Read Archive (SRA) under the BioProject accession number PRJNA1013789 (SRR25937770 and SRR25937769). The accession numbers of the genomes can be found in Supplementary Material 3 with the respective statistical data on the genome quality. The rpS3 genes are available under 10.6084/m9.figshare.25805215.v1.
